# Age-dependent endocrine and cellular adaptations to Mediterranean summer heat stress in lactating Saanen goats

**DOI:** 10.14202/vetworld.2026.29-38

**Published:** 2026-01-06

**Authors:** Fatma Atli, Sezgin Senturk

**Affiliations:** 1 Department of Animal Science, Faculty of Agriculture, Ege University, İzmir, Türkiye; 2 Department of Internal Medicine, Faculty of Veterinary Medicine, Bursa Uludağ University, Bursa, Türkiye

**Keywords:** dairy goats, endocrine adaptation, heat stress, HSP70 expression, milk yield, *Saanen* goats, thermoregulation, thyroid hormones

## Abstract

**Background and Aim:**

Heat stress (HS) substantially impairs dairy goat productivity in Mediterranean climates by disrupting metabolic, endocrine, and cellular homeostasis. High-yielding Saanen goats are particularly vulnerable because of elevated metabolic heat production, yet age-specific physiological responses to prolonged natural HS remain unclear. This study aimed to characterize age-dependent adaptations to progressive summer HS by evaluating changes in triiodothyronine (T3), thyroxine (T4), cortisol (CORT), and heat shock protein 70 (HSP70), and their relationship to daily average milk yield (DAMY). We hypothesized that increasing temperature–humidity index (THI) would suppress T3 and T4, moderately elevate CORT, and stimulate HSP70 expression, particularly in young goats.

**Materials and Methods:**

Thirty clinically healthy, lactating *Saanen* does were grouped into young, middle-aged, and old age groups (n = 10 per group). The study was conducted from May to August under natural Mediterranean field conditions. Ambient temperature, relative humidity, and THI were recorded daily. DAMY was measured automatically using a radio-frequency identification-linked milking system. Blood samples were collected twice monthly to quantify serum T3, T4, CORT, and HSP70 using commercial enzyme-linked immunosorbent assay kits. A repeated-measures general linear model evaluated the effects of age, month, and their interaction; significance was set at p < 0.05.

**Results:**

THI increased from “no HS” in May to “severe HS” in July and August, confirming sustained heat-load. DAMY declined from 2.59 ± 0.43 kg in May to 1.88 ± 0.40 kg in August. T4 decreased significantly in young and middle-aged goats, with the sharpest decline in middle-aged goats (92.96 to 61.82 nmol/L; p < 0.01). T3 also decreased significantly in young and middle-aged groups (p < 0.01), whereas older goats showed modest, nonsignificant reductions. CORT showed a mild, nonsignificant upward trend. HSP70 increased across all groups, with a significant rise in young goats (13.32 to 17.85 ng/mL; p < 0.05). T4 showed a strong positive correlation with DAMY (r = 0.78, p = 0.0027), whereas CORT showed a moderate negative correlation with DAMY (r = −0.58, p = 0.047).

**Conclusion:**

Lactating Saanen goats exhibit age-dependent dual adaptations to summer HS: endocrine suppression of thyroid activity, stronger in middle-aged goats, and cellular upregulation of HSP70, most evident in young goats. Monitoring T3, T4, CORT, HSP70, and DAMY can help identify thermally vulnerable life-stage groups and guide targeted cooling, nutritional, and breeding interventions in heat-stressed dairy systems.

## INTRODUCTION

Climate change and global warming have intensified the challenge of heat stress (HS) in livestock production, particularly in Mediterranean regions where prolonged summers expose animals to sustained thermal load. Goats are generally considered resilient ruminants; however, high-yielding dairy breeds, such as Saanen, are highly susceptible to HS because of their elevated metabolic demands and high milk production [1]. HS reduces feed intake, fertility, and milk yield while compromising immunity and overall welfare [2]. Understanding how endocrine and cellular systems respond to prolonged natural heat-load across different life stages of dairy goats is essential for maintaining productivity and welfare under warming climates [3].

Among the key endocrine regulators, the thyroid hormones triiodothyronine (T3) and thyroxine (T4) play central roles in basal metabolism and thermogenesis [4]. Suppression of T3 and T4 under HS is a well-documented adaptive mechanism that reduces endogenous heat production [5]. In parallel, activation of the hypothalamic–pituitary–adrenal (HPA) axis leads to the release of cortisol (CORT), a glucocorticoid critical for maintaining homeostasis and mobilizing energy reserves under stress [6]. Heat shock protein 70 (HSP70) functions as a molecular chaperone that protects against protein denaturation and oxidative damage at the cellular level, making it a robust biomarker of thermal strain [7]. Most previous studies have examined these markers separately or without distinguishing age classes, and relatively few have followed the same animals across an entire summer season under commercial farm conditions [8, 9].

Although HS in dairy goats has been extensively studied, significant gaps remain in understanding how different age groups respond to prolonged natural heat exposure at both endocrine and cellular levels. Existing studies have predominantly focused on single biomarkers, short-term heat challenges, or mixed-age populations, without stratifying physiological responses by life stage. Furthermore, most available evidence derives from controlled experimental settings rather than longitudinal observations under commercial Mediterranean farm conditions, where animals experience cumulative thermal load across the summer months. The concurrent evaluation of thyroid hormones (T3 and T4), CORT as an indicator of HPA axis activation, and HSP70 as a cellular chaperone response has rarely been conducted within the same study. Critically, the literature lacks comprehensive investigations that integrate endocrine and molecular markers with production traits such as daily average milk yield (DAMY) across age-specific cohorts. As a result, knowledge of age-dependent physiological plasticity, vulnerability, and adaptive capacity in heat-stressed dairy goats remains limited, hindering the development of life-stage–tailored management strategies for improving resilience under warming climates.

Therefore, this study aimed to characterize age-dependent endocrine and cellular adaptations in lactating Saanen goats exposed to progressive Mediterranean summer HS. Specifically, the study evaluated monthly changes in T3, T4, CORT, and HSP70 concentrations in young, middle-aged, and old goats and examined their associations with DAMY under natural field conditions. By simultaneously assessing these complementary physiological markers during sustained HS, the study sought to identify distinct thermoregulatory strategies across life stages and to determine which age groups exhibit greater endocrine sensitivity or cellular resilience. Ultimately, the aim was to provide a comprehensive physiological framework that supports age-specific HS mitigation strategies and enhances understanding of thermotolerance mechanisms in dairy goats managed under real farm environments.

## MATERIALS AND METHODS

### Ethical approval

All procedures involving animals in this study were conducted in strict accordance with institutional, national, and international regulations governing the ethical use of animals in research. The experimental protocol was reviewed and approved by the Ege University Local Animal Experiments Ethics Committee (Ege Üniversitesi Hayvan Deneyleri Yerel Etik Kurulu – HADYEK), Izmir, Turkey (Approval No. 2020-082; March 2020). Approval was granted following a comprehensive assessment of the study design, animal handling procedures, housing conditions, sampling frequency, and potential welfare impacts.

Throughout the study, all goats were monitored daily by trained farm personnel and a veterinarian to detect early signs of stress, discomfort, or health complications. Blood sampling was performed by the same experienced veterinarian using minimal manual restraint to minimize handling-related stress. No invasive procedures beyond standard jugular venipuncture were conducted, and animals experienced no prolonged or severe distress. Environmental conditions, including ventilation, shade availability, and access to clean water, were continuously maintained to meet welfare standards, especially during periods of high temperature–humidity index (THI).

This research complied with Directive 2010/63/EU of the European Parliament on the protection of animals used for scientific purposes and with the European Convention for the Protection of Vertebrate Animals Used for Experimental and Other Scientific Purposes. Reporting followed the Animal Research: Reporting of In Vivo Experiments 2.0 guidelines to ensure transparency, reproducibility, and ethical accountability. All animals remained the property of the research farm, were returned to routine management after the study, and were not euthanized or subjected to any terminal procedures as part of the research.

### Study period and location

The 4-month experimental study (May–August 2021) was conducted at the Faculty of Agriculture, Farm Animal Production, Research, and Application Center, Ege University, Izmir, Turkey. Izmir, on the Aegean coast, has a Mediterranean climate with hot, dry summers. Although the core summer months are June–August, animals in this region are exposed to high temperatures for 4–5 months beginning in May; therefore, May–August was selected to capture the onset, peak, and late phases of seasonal HS.

Daily ambient temperature (AT; °C) and relative humidity (RH; %) were recorded using a digital thermo-hygrometer (Hasvet Medical, Antalya, Türkiye) positioned at animal height (~1.5 m). Measurements were taken three times per day (08:00, 14:00, 20:00), and daily averages were used to calculate the THI using the following equation:

THI = T – (0.31 – 0.31 × RH) × (T – 14.4)

Where, T = AT (°C) and RH = RH (%)

Heat-load categories were defined as follows [10]:

- No HS: THI < 22.3
- Mild HS: 22.3 ≤ THI < 23.3
- Moderate HS: 23.3 ≤ THI < 25.6
- Severe HS: THI ≥ 25.6.

### Animals and experimental design

Thirty clinically healthy Saanen goats from the Ege University research farm were enrolled. All animals received routine herd health care, including annual vaccinations and regular veterinary monitoring. Only nonpregnant does were included.

Goats were assigned to three age groups (n = 10 per group):

- Young: 1–2 years, parity 1–2
- Middle-aged: 3–4 years, parity 3–4
- Old: ≥5 years, parity ≥5

At the start of the study (May), the average days in milk (DIM) for young, middle-aged, and old goats were ~70, 80, and 85 days, respectively. DIM increased by ~30 days per month, reaching ~160–175 days by August, indicating that all animals remained in mid-lactation. Monthly body weight (BW) and parity records were retrieved from farm records to assess group-wise BW changes under progressive HS (PHS).

### Housing and management

The barn had an internal height of 2.5 m and consisted of four lactating-goat pens (96 m² each; total 384 m²). The gable roof was constructed from galvanized sheets, with internal heights ranging from 6 m (peak) to 4 m (sides). Each of 12 pens had a 10 m² outdoor exercise area (total 120 m²).

Goats were managed in a semi-open system that integrated shaded concrete pens and outdoor exercise yards. Indoor resting space provided approximately 1.5–2.0 m² per animal. Deep-litter bedding and concrete floors were cleaned daily. Ventilation was provided by open sidewalls, ridge openings, and mechanical ventilation systems. Goats had access to shaded outdoor areas during the day and returned indoors for milking and overnight housing. Automatic waterers supplied clean drinking water *ad libitum*.

### Feeding and nutrition

All goats received the same total mixed ration throughout the study, consisting of:

- 0.8 kg of pelleted concentrate
- 2.0 kg of corn silage
- 0.2 kg of wheat straw
- 0.6 kg of alfalfa hay

Individual allotments were controlled using a radio-frequency identification (RFID)-based feeding system. Feeding occurred twice daily after milking. Water and mineral blocks were available *ad libitum*. The diet’s chemical composition, including dry matter, crude protein, neutral detergent fiber, acid detergent fiber, and metabolizable energy, was analyzed using standard Weende and Van Soest methods.

### Milk yield recording

DAMY; kg/day) was automatically recorded using an electronic milking system integrated with RFID ear tags. Morning and evening yields were summed to calculate DAMY. Monthly averages per goat were used to evaluate seasonal trends and to assess relationships between DAMY, DIM, and endocrine/cellular markers.

### Blood sampling and serum preparation

Blood samples (2 mL) were drawn from the jugular vein twice monthly (~every 15 days) between 08:30 and 09:30 h to minimize circadian variability, particularly for CORT. Samples were collected without anticoagulant, allowed to clot, and centrifuged at 3,000 rpm (approximately 1,200 × g) for 10 min at 4°C. Serum aliquots were stored at −20°C until analysis of T3, T4, CORT, and HSP70. Storage duration did not exceed 5 months, and repeated freeze–thaw cycles were avoided.

### Biochemical analyses

Serum T3, T4, CORT, and HSP70 concentrations were quantified using commercial ELISA kits (Everon Life Science Co., New Delhi, India): Goat T3 ELISA Kit (Cat. No: EA0027Go), Goat T4 ELISA Kit (Cat. No: EA0011Go), Goat CORT ELISA Kit (Cat. No: EA0007Go), and Goat HSP70 ELISA Kit (Cat. No: E0057Go). All samples were run in duplicate, with mean values used for statistical analysis. Calibration curves were constructed for each assay, and internal quality-control sera were included. Sensitivities and detection ranges matched physiological caprine values. Intra- and inter-assay coefficients of variation were <10% and <15%, respectively; plates failing quality-control criteria were repeated.

### Statistical analysis

All analyses were performed using SPSS Version 26.0 (IBM Corp., Armonk, NY, USA). Continuous variables (T3, T4, CORT, HSP70, and DAMY) were assessed for normality using the Kolmogorov–Smirnov and Shapiro–Wilk tests; homogeneity of variance was evaluated with Levene’s test.

Endocrine and cellular markers were analyzed using a general linear model (GLM) with age-group (young, middle-aged, old) and month (May–August) as fixed factors. The age × month interaction was tested. Goat ID was included as a random factor in the repeated-measures model. When the interaction was nonsignificant, main effects were interpreted. Tukey’s Honestly Significant Difference test was used for post hoc comparisons (*p* < 0.05).

DAMY was analyzed using repeated-measures analysis of variance, with month as the within-subject factor and age as the between-subject factor. Sphericity was assessed using Mauchly’s test, and the Greenhouse–Geisser correction was applied when appropriate.

Pearson correlation analysis assessed associations between DAMY and physiological markers. Correlation coefficients (r), p-values, and biological interpretations were reported. Statistical significance was defined as p < 0.05, and 0.05 ≤ p < 0.10 was considered a biological trend. Descriptive statistics are presented as mean ± standard error of the mean (SEM), with standard deviation calculated as SEM × √n.

## RESULTS

### Climatic conditions and THI

Descriptive climatic data showed a progressive rise in heat-load from May to August. The lowest daily maximum temperature increased from 25.11°C in May to 39.48°C in August, representing a nearly 14°C rise. Mean monthly temperatures were 21.50°C, 27.18°C, 31.82°C, and 30.81°C in May, June, July, and August, respectively, while mean RH averaged 40.65%. As summer progressed, the THI increased from moderate to severe HS categories, and monthly differences in maximum temperature, AT, average humidity, and THI were statistically significant (*p* < 0.05), confirming sustained HS throughout the study. RH declined from May (54.9%) to July (38.2%), reflecting the typical arid summer pattern of the Mediterranean region.

THI showed a highly significant month effect (p < 0.0001), increasing from “no HS” in May (20.4) to “moderate HS” in June (24.8) and “severe HS” in July and August (28.5 and 27.9, respectively). These findings confirm that goats experienced persistent and intensifying HS throughout the experimental period (Table 1).

**Table 1:** Descriptive statistics (mean values) of the climatic variables recorded from May to August.

Month	Mean maximum temperature (°C)	Mean minimum temperature (°C)	Mean AT (°C)	Mean relative humidity (%)	Mean THI
May	25.11	17.17	21.50	54.92	20.44
June	31.68	22.18	27.02	46.40	24.84
July	37.42	27.17	31.82	38.16	28.51
August	39.91	24.43	31.08	40.28	27.92

All climatic variables showed a significant month effect (p < 0.05 for humidity; p < 0.0001 for temperature and THI). AT = Ambient temperature, RH = Relative humidity. Mean maximum temperature (°C): The average of daily maximum ATs recorded each month. Mean minimum temperature (°C): The average of daily minimum ATs recorded each month. Mean AT (°C): The monthly mean of daily temperature measurements. Mean relative humidity (%): The average monthly relative humidity, measured as the daily mean percentage. Temperature–Humidity Index (THI): THI was calculated to quantify the combined heat and humidity stress experienced by the animals. Temperature variables: p < 0.0001; relative humidity: p < 0.05; THI: p < 0.0001

### BW and milk yield (DAMY)

BW remained stable within each age-group, with minor fluctuations (<5 kg), indicating maintenance of overall body condition despite progressive HS.

DAMY declined across all age groups. Mean DAMY decreased from 2.59 ± 0.43 kg/day in May to 2.30 ± 0.39 kg/day in June, 2.11 ± 0.40 kg/day in July, and 1.88 ± 0.40 kg/day in August. Young goats produced 2.69, 2.41, 2.24, and 1.97 kg/day; middle-aged goats produced 2.69, 2.47, 2.27, and 2.06 kg/day; and old goats produced 2.40, 2.01, 1.84, and 1.60 kg/day from May to August, respectively (Table 2).

**Table 2:** Monthly changes in DAMY in *Saanen* goats according to age.

Month	Young (kg/day)	Middle-aged (kg/day)	Old (kg/day)	Overall mean ± SD (kg/day)
May	2.69	2.69	2.40	2.59 ± 0.43
June	2.41	2.47	2.01	2.30 ± 0.39
July	2.24	2.27	1.84	2.11 ± 0.40
August	1.97	2.06	1.60	1.88 ± 0.40

Values for age groups represent the monthly mean DAMY derived from individual goat measurements. DAMY = Daily average milk yield, SD = Standard deviation. Overall mean ± SD values for all goats, pooled across age groups, for each month.

Repeated-measures analysis confirmed a significant month effect on DAMY (p < 0.05), with no strong age × month interaction, indicating similar seasonal declines across age groups. DAMY decreased significantly over the season (F_3,87_ = 141.58, p < 0.0001), indicating that the reduction was due to PHS rather than random variation.

### Thyroid hormones (T3 and T4)

Clear endocrine adaptations to summer HS were observed. T4 concentrations declined across all age groups, with the largest reduction in middle-aged goats (from 92.96 nmol/L in May to 61.82 nmol/L in August; p < 0.01). Young and old goats showed decreasing trends, although the reductions in old goats were not significant. GLM analysis showed significant effects of month and age on T4 (p < 0.05), with a notable age × month interaction driven by the pronounced decline in middle-aged goats.

T3 showed a similar pattern. Young and middle-aged goats exhibited significant decreases in T3 across the summer months (p < 0.01), particularly in July and August, whereas the decline in old goats was mild and nonsignificant. These reductions reflect heat-induced downregulation of thyroid activity, a known mechanism for reducing endogenous heat production. Detailed values are shown in Table 3.

**Table 3:** Age-month-related hormonal effects of heat stress in *Saanen* goats.

Animals	Variable	T4 (nmol/L)	T3 (ng/ml)	CORT (ng/ml)	HSP70(ng/mL)
Young Animals (n = 10)	May	87.79	4.08^a^	3.94	13.32^b^
	June	81.47	3.66^ab^	4.48	15.16^ab^
	July	79.30	3.24^bc^	4.49	15.49^ab^
	August	65,03	3.14^c^	5.09	17.85^a^
	SEM	7.10	0.12	0.55	0.97
	SD	22.45	0.38	1.74	3.07
	p-value	0.16	<0.01	0.54	0.03

Middle age Animals (n = 10)	May	92.96^a^	3.77^a^	4.12	12.73
	June	80.95^ab^	3.48^ab^	4.58	13.94
	July	71.64^ab^	3.19^bc^	4.84	15.05
	August	61.82^b^	2.97^c^	5.25	15.73
	SEM	5.58	0.13	0.49	0.93
	SD	17.65	0.41	1.55	2.94
	p-value	<0.01	<0.01	0.43	0.15

Old Animals (n = 10)	May	79.87	3.97	4.60	11.61
	June	85.08	3.67	4.79	13.10
	July	72.50	3.57	4.43	13.74
	August	62.77	3.3	4.65	14.34
	SEM	9.34	0.23	0.66	1.36
	SD	29.54	0.73	2.09	4.30
	p-value	0.38	0.3	0.99	0.56

T4 = Thyroxine, T3 = Triiodothyronine, CORT = Cortisol, HSP70 = Heat Shock Protein 70, SEM = Standard error of the mean, SD = Standard deviation, GLM = General linear model. Values represent means for each age-group (young, middle-aged, and old) across four consecutive summer months (May–August). Different superscript letters (a, b, c) within the same column indicate statistically significant differences among months for that age-group (p < 0.05). “a” = highest value; “c” = lowest value. Values sharing the same letter are not significantly different. SEM denotes the standard error of the mean, reflecting within-group variation across animals. Based on a one-way GLM analysis, p-values refer to the effect of month within each age category. Values are presented as mean ± SE ± SD for each physiological parameter. The SE values were taken from the original GLM outputs; the SD values were calculated using the formula SD = SE × √n, with n = 10 goats per age-group.

### CORT

CORT levels tended to increase toward peak summer months, suggesting activation of the HPA axis under thermal load. However, the effects of month, age, and their interaction were not statistically significant (p > 0.05), reflecting high interindividual variability (Table 3). All CORT concentrations remained within physiological ranges. These findings support the view that CORT is a more variable HS indicator than T3, T4, or HSP70 under field conditions.

### HSP70

HSP70 concentrations increased progressively from May to August. Young goats showed a significant rise from 13.32 ng/mL in May to 17.85 ng/mL in August (p < 0.05). Middle-aged and old goats also showed upward trends, though these were nonsignificant. The GLM indicated a significant month effect and a tendency for an age × month interaction, driven by the stronger HSP70 response in young goats. These findings suggest greater cellular chaperone activity and stress resilience in younger animals.

### Integrated endocrine, cellular, and production responses

The integrated results revealed a consistent physiological response to HS in Saanen goats: (1) reductions in T3 and T4, (2) modest and variable increases in CORT, (3) elevated HSP70 levels—particularly in young goats—and (4) a progressive decline in DAMY over the summer (Table 3).

Exploratory analyses suggested that goats with lower T3/T4 tended to show reduced DAMY, whereas higher HSP70 was associated with greater thermal strain. Correlation patterns reflected the complexity of physiological responses during HS (Table 4).

**Table 4:** Pearson correlation coefficients between DAMY and endocrine/cellular stress markers in *Saanen* goats.

Variable	r (with DAMY)	p-value	Interpretation
T4	0.78	0.0027	Strong positive, significant
T3	0.57	0.0534	Moderately positive, borderline (trend)
CORT	–0.58	0.0474	Moderate negative, significant
HSP70	–0.36	0.2466	Weak negative, not significant

DAMY = Daily average milk yield, T3 = Triiodothyronine, T4 = Thyroxine, CORT = Cortisol, HSP70 = Heat shock protein 70. Positive r-values indicate that milk yield increases with increasing hormone concentration, whereas negative values indicate an inverse association. Statistical significance was set at p < 0.05.

Correlation analysis based on age-group × month means (n = 12) showed:

- T4 was strongly and positively correlated with DAMY (r = 0.78, p = 0.0027).
- CORT was moderately and negatively correlated with DAMY (r = –0.58, p = 0.047).
- T3 was moderately correlated with DAMY (r = 0.57, p = 0.053).
- HSP70 was negatively correlated with DAMY, but the correlation was nonsignificant (r = –0.36, p = 0.25).

Higher thyroid activity was therefore associated with superior lactational performance, whereas increased HPA-axis activation was associated with lower productivity under HS.

## DISCUSSION

### Overview of endocrine and cellular adaptations to HS

This study assessed the endocrine and cellular responses of Saanen goats exposed to progressive natural summer heat, emphasizing age-dependent patterns in T3, T4, CORT, and HSP70 and their associations with DAMY. By monitoring the same goats over four consecutive Mediterranean summer months and differentiating three age groups, the study provides novel insight into life-stage–specific thermoregulatory strategies in high-producing dairy goats.

### Age-dependent thyroid suppression under HS

Marked reductions in thyroid hormones were observed in young and middle-aged goats, consistent with previous reports showing that HS suppresses thyroid activity to reduce metabolic heat production [5, 8]. In middle-aged goats, T4 declined by approximately one-third from May to August, the greatest reduction across age groups, suggesting that animals at peak lactation may rely heavily on thyroid downregulation as an energy-saving strategy under sustained HS. Older goats showed smaller, nonsignificant reductions, likely due to lower baseline metabolic and lactational demands that limit further suppression.

Similar age-dependent decreases in T3 and T4 have been reported in goats and other small ruminants, particularly in high-producing or mid-lactation animals [1, 2, 11]. These findings support the view that thyroid suppression is a conserved endocrine mechanism that reduces endogenous heat production and conserves energy and water during thermal stress.

### CORT variability and HPA-axis ınvolvement

CORT concentrations showed an upward trend toward the hottest months but were highly variable, with no significant effects of month or age-group. This pattern mirrors findings from other field studies, in which single-time-point CORT measurements often show large interindividual variation and inconsistent statistical significance during HS [12, 13]. CORT is strongly influenced by circadian rhythm, acute handling, and individual stress-coping capacity, all of which likely contributed to the observed variability.

The interaction between the HPA-axis and the hypothalamic–pituitary–thyroid axis is relevant. CORT can suppress thyrotropin-releasing hormone and thyroid–stimulating hormone, thereby reducing T3 and T4 synthesis [14–16]. Thus, even a modest increase in CORT may have contributed to declines in thyroid hormones. Future studies incorporating serial CORT sampling or metrics such as heart rate variability could provide a more accurate assessment of HPA responses to HS.

### HSP70 ınduction and cellular stress responses

HSP70 increased across all age groups, with the most pronounced rise in young goats. This strong induction suggests a more vigorous cellular protective response in younger animals, consistent with evidence that younger organisms possess greater proteostatic capacity and enhanced stress-induced chaperone responses [6, 9]. In contrast, middle-aged and older goats showed smaller increases in HSP70, which may indicate reduced cellular plasticity or reliance on endocrine rather than cellular mechanisms for thermal adaptation.

Previous studies in goats and cattle identified HSP70 as a sensitive biomarker of thermal load, showing elevated expression during acute and chronic HS [6, 13, 17]. The present findings extend this literature by demonstrating age-dependent differences in serum HSP70 responses during natural summer HS and by integrating these results with endocrine and production traits.

### Proposed age-dependent two-tier adaptation model

Based on combined endocrine, cellular, and production data, we propose an age-dependent two-tier adaptation framework for HS in Saanen goats.

- Young goats: Strong HSP70 induction and moderate thyroid suppression suggest that cellular defense mechanisms dominate their thermotolerance strategy. Their robust proteostatic capacity may help maintain physiological function despite high THI.
- Middle-aged goats: The most pronounced T3/T4 suppression and only modest HSP70 increases indicate reliance on endocrine modulation to reduce metabolic heat-load. As the highest-producing cohort, they appear more sensitive to systemic endocrine adjustments.
- Older goats: Relatively stable T3/T4 and HSP70 profiles may reflect lower metabolic activity and limited adaptive reserves, resulting in weaker responses.

This crossover pattern, in which cellular defense is dominant in young goats and endocrine adjustment is dominant in middle-aged goats, highlights that HS adaptation is not uniform across age groups. These distinctions are important for developing age-specific management strategies and selecting heat-resilient genotypes [18].

### Production consequences and practical ımplications

As expected, DAMY declined from May to August, mirroring the increase in THI. Although correlations between DAMY and physiological markers were modest, goats with greater thyroid suppression or higher HSP70 tended to show greater production losses, indicating heightened thermal strain [19].

#### From a management perspective:

- Middle-aged, high-yielding goats may require priority cooling interventions (e.g., shade structures, fans, misting) and nutritional support, such as increased dietary energy and antioxidants.
- Young goats with strong HSP70 responses may benefit from monitoring to prevent chronic cellular stress that could compromise long-term performance.
- Routine monitoring of T3, T4, CORT, HSP70, and DAMY can help identify at-risk animals early and guide targeted interventions.

### Strengths, limitations, and future directions

Key strengths of the study include its four-month longitudinal design, integration of endocrine, molecular, and production data, and focus on age-specific responses under authentic field conditions. Nonetheless, some limitations warrant consideration:

- CORT was measured at a single morning time point; additional sampling or hair CORT could better represent HPA activity.
- HSP70 was measured only in serum; tissue-level or transcriptional analyses could provide deeper insight into cellular pathways.
- Although DIM was well controlled and BW remained stable, larger sample sizes or multifarm studies would enhance generalizability.

Future research should use multi-omics approaches (e.g., transcriptomics, metabolomics) to identify molecular networks underlying heat resilience and determine whether T4 suppression or HSP70 induction serves as a reliable phenotypic marker for breeding thermotolerant lines. Controlled studies manipulating THI, along with behavioral and feed-intake monitoring, would clarify causal relationships between physiological changes and production outcomes.

## CONCLUSION

This study demonstrated that lactating Saanen goats exhibit distinct age-dependent endocrine and cellular adaptations when exposed to progressive Mediterranean summer heat. Thyroid hormones (T3 and T4) declined significantly in young and middle-aged goats, with middle-aged animals showing the steepest T4 suppression, from 92.96 nmol/L in May to 61.82 nmol/L in August, reflecting strong endocrine downregulation to reduce metabolic heat production. Young goats displayed a pronounced increase in HSP70, rising from 13.32 ng/mL to 17.85 ng/mL, indicating a vigorous cellular chaperone response. In contrast, older goats showed smaller, nonsignificant changes in both hormone and HSP70 concentrations. DAMY decreased steadily across all age groups, in parallel with the increasing THI, confirming the detrimental influence of prolonged HS on lactational performance. Correlation analyses further revealed that higher T4 strongly predicted greater DAMY (r = 0.78), whereas elevated CORT correlated negatively with milk yield (r = −0.58), underscoring the interplay between endocrine status and production outcomes during HS.

From a practical perspective, these findings underscore the need for age-specific heat-mitigation strategies in dairy goat production systems. Middle-aged, high-producing goats may benefit from prioritized cooling interventions (e.g., shade structures, fans, misting systems) and dietary adjustments to support metabolic adaptation, whereas young goats exhibiting strong HSP70 responses may require monitoring to prevent prolonged cellular stress. Routine evaluation of T3, T4, CORT, HSP70, and DAMY offers a feasible approach for identifying goats most vulnerable to HS and implementing timely management actions.

Overall, this work proposes an age-related two-tier thermoregulatory model in which young goats rely more on cellular defenses and middle-aged goats depend predominantly on endocrine suppression to cope with HS. Understanding these life-stage–specific adaptations is essential for enhancing resilience, maintaining productivity, and guiding breeding and management decisions in heat-challenged dairy goat systems. Future research using multi-omics tools, controlled HS trials, and larger herd datasets will help refine biomarker-based selection strategies and further elucidate the physiological foundations of thermotolerance.

## DATA AVAILABILITY

All the generated data are included in the manuscript.

## AUTHORS’ CONTRİBUTİONS

FA and SS: Conceptualization, study design, and drafted the manuscript. FA: Data collection, animal management, and laboratory analyses. SS: Statistical analysis and revised the manuscript. Both authors have read and approved the final version of the manuscript.
